# Relationship between medication safety‐related processes and medication use in residential aged care facilities

**DOI:** 10.1111/ajag.13352

**Published:** 2024-06-24

**Authors:** Ramesh Sharma Poudel, Kylie A. Williams, Lisa G. Pont

**Affiliations:** ^1^ Discipline of Pharmacy, Graduate School of Health University of Technology Sydney Sydney 2007 New South Wales Australia

**Keywords:** correlation of data, drug utilisation, long‐term care, patient safety

## Abstract

**Objective:**

To explore the association between the implementation of medication safety‐related processes measured with the Medication Safety Self‐Assessment for Long‐Term Care (MSSA‐LTC) tool and medication use in residential aged care facilities (RACFs).

**Methods:**

A descriptive cross‐sectional study was conducted in Australian RACFs. Data on facility characteristics, aggregated medication use at the facility level for selected medications commonly associated with a high risk of harm and the MSSA‐LTC were completed by clinical pharmacists providing clinical pharmacy services. The Spearman's correlation test was used to evaluate the association between the MSSA‐LTC score and medication use. A scatter plot between the MSSA‐LTC score and medication use data was generated, and a linear trend line was plotted using the least squares method.

**Results:**

Data were collected from 31 RACFs servicing 2986 residents. Most medication safety‐related processes were implemented in Australian RACFs. A higher facility MSSA‐LTC score was associated with a lower proportion of residents with polypharmacy (*r* = −.48, *p* = .01) and one or more benzodiazepines (*r* = −.41, *p* = .03). In addition, a negative linear trend was observed between the MSSA‐LTC score and the average number of medications per resident, the proportion of residents with one or more anticonvulsants and the proportion of residents using one or more opioid analgesics.

**Conclusions:**

This study indicates that implementing medication safety‐related processes may improve medication use in RACFs.

**Policy Impact**

This study found that residential aged care facilities (RACFs) with a higher Medication Safety Self‐Assessment for Long‐Term Care (MSSA‐LTC) tool score most likely have a lower proportion of residents with polypharmacy and benzodiazepines. A higher level of implementation of medication safety‐related processes included in the MSSA‐LTC tool may improve medication use in RACFs.

## 1 INTRODUCTION

Medication‐related harm is a major safety concern in residential aged care facilities (RACFs).^1^ Frailty, medical complexity, high levels of medication use and high incidence of medication errors all increase the risk of medication‐related harm for residential aged care residents.^2^, ^3^, ^4^ Studies have indicated that 70% of all residential aged care residents experience at least one medication error,^5^, ^6^ and half are exposed to potentially inappropriate medicines.^7^ The incidence rates of medication‐related harm in RACFs range from 1.89 to 36.4 per 100 resident‐months,^1^, ^8^ with an annual estimated cost of $ 7.6 billion in the United States alone.^9^ The most common medication‐related harm reported in residential aged care residents were bleeding, thromboembolic events, hypoglycaemia, falls and constipation.^8^

Multiple factors contribute to medication‐related harm in residential aged care settings.^8^ Many of these factors have been linked to ‘latent failures’ (e.g., system or process failures) in medication‐related processes, including inadequate nursing staff medication knowledge and training, lack of interprofessional collaboration, availability of limited resident information for prescribers, lack of health information technology, workload and time pressure for staff and staff distraction during medication administration.^5^, ^8^, ^10^

In Australia, guiding principles for medication management in RACFs outline 15 principles to support safe medication management at the facility level.^11^ These cover areas such as information resources for staff and residents, continuity in medication supply, medication administration, and medication storage and disposal. The Australian Aged Care Quality and Safety Commission, a national independent accreditation body, regularly audits the RACFs to ensure that they meet the quality standards (eight quality standards) for residential aged care, including safe medication management.^12^ However, no medication safety assessment tool is available to enable Australian RACFs to self‐assess the level of implementation of medication safety‐related processes and to identify the potential failures and opportunities for improving medication safety similar to the Australian hospitals.^13^

The Medication Safety‐Self Assessment for Long‐Term Care (MSSA‐LTC) tool was developed by the Institute for Safe Medication Practices Canada (ISMP Canada) to assess the implementation of medication safety‐related processes in RACFs.^14^ The MSSA‐LTC tool version II comprises 129 items divided into 10 key elements: resident information, drug information, communication, drug labelling and packaging, drug storage and distribution, medication delivery devices, environmental factors, staff competence and education, resident education and quality processes and risk management. Each item relates to a medication‐related process considered important for medication safety. The MSSA‐LTC tool is designed for self‐assessment of the level of implementation of medication safety‐related processes to enable health professionals and facility staff to identify suboptimal processes or ‘latent failure’ in the residential aged care medication‐related processes as part of continuous quality improvement processes. In the MSSA‐LTC, the level of implementation of each item is assessed using a 5‐response Likert‐type scale with response options ranging from ‘no activity to implement an item’ to ‘fully implemented throughout the facility’ or ‘not applicable’, which are then converted to a numerical score (0–4). Higher total MSSA‐LTC scores and MSSA‐LTC key element scores are interpreted as reflecting well‐implemented and established (more mature) medication safety‐related processes. The tool is routinely used in Canadian RACFs to assess their medication‐related processes objectively, implement quality improvement initiatives and evaluate their efforts over time. Our research team conducted a study to determine the validity (content and face) of using the MSSA‐LTC tool in Australian RACFs using the RAND Appropriateness Method^15^ and found that the MSSA‐LTC tool was suitable for use in Australian RACFs (unpublished data). A comparative analysis of RACFs across seven countries (Australia, Canada, Japan, New Zealand, Switzerland, the UK and the United States) found considerable similarities between aged care systems in Australia and Canada,^16^ which may account for the suitability of the Canadian MSSA‐LTC tool in Australia.

An older adult has substantial comorbidity and typically takes many medications. It has been estimated that 91% of all residents use five or more medications concomitantly,^17^ and on average, residents use up to 11 different medicines.^18^ Psychotropic medicines (antipsychotics, hypnotics/sedatives and antidepressants), antibiotics and opioid analgesics are among the most commonly prescribed medications in residential aged care residents,^18^, ^19^, ^20^, ^21^ and these medicines are also commonly implicated in medication‐related harm.^22^, ^23^ Considerable variation has been observed in the numbers and extent of use of these medicines across the RACFs.^24^, ^25^ Previous research has reported that resident or facility characteristics only predicted a small proportion of variation in medicine use across RACFs.^26^, ^27^ Studies in hospital settings have shown that well‐implementation of patient safety‐related processes was associated with fewer medication errors as well as other adverse events.^28^, ^29^ While there is evidence supporting that better safety processes improve medication management in the hospital setting, no research has explored the association between the implementation of medication safety‐related processes and medication use in RACFs. Therefore, the objective of this study was to explore the association between the implementation of medication safety‐related processes measured with the MSSA‐LTC tool and medication use in RACFs.

## 2 METHODS

### 2.1 Study design and setting

A descriptive cross‐sectional study design was used to explore the association between the implementation of medication safety‐related processes and medication use in RACFs between November 2022 and February 2023. The study was conducted in Australian RACFs. In Australian RACFs, medications are prescribed by GPs who visit the residential aged care periodically as required but are not available onsite. Each facility will usually contract a single off‐site community pharmacy to supply medicines to the residents within that facility based on traditional prescriptions or the National Residential Medication Chart. The majority of residents are dependent on staff for medication administration. A separate clinical pharmacist is contracted to provide clinical services such as comprehensive medication review.^30^

### 2.2 Participants and recruitment

Pharmacists providing clinical or medication review services to one or more Australian RACFs were recruited using a convenience sampling approach. Two recruitment strategies were used. First, pharmacists belonging to a social media network for pharmacists providing clinical pharmacy services to RACFs were invited to participate in the study. The second strategy was to disseminate the invitation via one of Australia's largest providers of pharmacist clinical services to RACFs. Pharmacists interested in participating in the study were provided with the research team's contact details. All participants received written participant information, and informed consent was obtained prior to participation in the study. Pharmacists were not compensated financially for their participation. However, all participating pharmacists received access to the MSSA‐LTC tool for 12 months. Recruitment continued until the required sample size was met.

### 2.3 Data collection

Each participating pharmacist completed an online data collection form for one or more RACFs for which they provided clinical or medication review services. The data collection form included information on facility characteristics, medication use characteristics and the pharmacist's perceived level of implementation of each of the 129 MSSA‐LTC items in the relevant facility. These data were collected electronically using REDCap.^31^

#### 2.3.1 Facility characteristics

Data collected on facility characteristics included facility size, location, rurality (based on the 2019 Modified Monash Model Remoteness category by postcode^32^), ownership type (government, private and not‐for‐profit) and the number of residents who had received a medication review over the past 12 months.

#### 2.3.2 Medication use characteristics

Aggregated medication use data collected for each facility included the mean number of medications prescribed per resident, the total number of residents with polypharmacy (nine or more medications)^33^ and the number of residents prescribed each of the following medication classes: antipsychotics, antidepressants, systemic antibiotics, benzodiazepines, opioid analgesics and anticonvulsants. We considered both regular and *prn* medications while collecting medication use data. We chose these classes of medications as they are commonly prescribed in residential aged care,^18^, ^19^, ^20^, ^21^ widely involved in medication‐related harm^22^, ^23^ and considerable variation in the extent of use across RACFs has been identified.^24^, ^25^

#### 2.3.3 Medication‐safety related processes

The level of implementation of medication safety‐related processes was measured using the MSSA‐LTC tool developed by ISMP Canada. Participating pharmacists were provided with detailed written instructions on answering each MSSA‐LTC item. For each of the 129 MSSA‐LTC tool items, the pharmacist was asked to record the item's implementation level. Numeric values between 0 and 4 were allocated to each response option with 0 for ‘no activity to implement an item’, 1 for ‘discussed but not implemented’, 2 for ‘partially implemented in some or all areas’, 3 for ‘fully implemented in some areas’ and 4 for ‘fully implemented throughout’ or ‘not applicable item’.

### 2.4 Sample size

The study was powered to detect a univariate association via a moderate correlation (*r* > .6) between the MSSA‐LTC score and each medication use characteristic with an alpha of .05 and 80% power.^34^ A sample of 20 RACFs would allow a moderate correlation to be detected. To allow for incomplete data and improve the power of the study, an additional 10 facilities were proposed to be recruited, giving a sample size of 30 facilities.

This study was approved by the Human Research Committee of the University of Technology Sydney (ETH22‐7358).

### 2.5 Data analysis

Descriptive statistics were used to summarise the facility's characteristics and medication use characteristics. The proportion of residents with polypharmacy, one or more antipsychotics, antidepressants, antibiotics, benzodiazepines, opioid analgesics and anticonvulsants was calculated for each facility as the total number of residents prescribed the relevant medication class divided by the total number of residents in the facility. The total MSSA‐LTC score per facility was calculated by summing the scores assigned to each item.^14^ Where pharmacists did not provide an answer for the level of implementation of a particular MSSA‐LTC item, it was treated as missing data and half‐scale imputation from the total item score was used.^35^

The Spearman's correlation was used to examine the association between the MSSA‐LTC score and the proportion of residents prescribed with polypharmacy, the average number of medications per resident, one or more antipsychotics, antidepressants, antibiotics, benzodiazepines, opioid analgesics and anticonvulsants. A scatter plot between the MSSA‐LTC score and medication use data was generated, and a linear trend line was plotted using the least squares method. A *p*‐value of <.05 was considered as significant. Data were analysed using Microsoft Excel and SPSS v.20 (IBM Corp., Armonk, NY).

## 3 RESULTS

### 3.1 Characteristics of RACFs

Nineteen pharmacists completed data for 31 RACFs servicing 2986 residents. More than half of the RACFs were from New South Wales (*n* = 17, 55%), most were from metropolitan areas (*n* = 23, 74%), and about two‐thirds of facilities were privately owned (*n* = 21, 68%). The majority of the facilities were medium‐sized, with the median (interquartile range) number of residents per facility being 95 (60–130). On average, over half of residents (53%) had received a formal medication review by a pharmacist in the past 12 months (Table 1).

**TABLE 1:** Description of facility demographic and medicine use (n=31).

		*N* (%), *unless stated*
Location	New South Wales	17 (55)
	Queensland	1 (3)
	South Australia	9 (29)
	Victoria	4 (13)
Remoteness	Metropolitan areas	23 (74)
	Regional centres	2 (7)
	Larger rural towns	2 (7)
	Small rural towns	4 (13)
Ownership type	Government	3 (10)
	Private	21 (68)
	Not‐for‐profit	7 (23)
Size	Median (IQR) number of residents per facility	95 (60–130)
Medication review	Median (IQR) proportion of residents per facility with a pharmacist medication review in the past 12 months	53% (41%–84%)
Medication use	Median (IQR) of the average number of medicines per resident for each facility	13 (12–14)
	Median (IQR) proportion of residents per facility with polypharmacy	53% (34%–60%)
	Median (IQR) proportion of residents per facility using one or more:	
	Antipsychotics	19% (15%–32%)
	Antidepressants	42% (33%–48%)
	Antibiotics	13% (9%–27%)
	Benzodiazepines	26% (17%–31%)
	Opioid analgesics	34% (23%–42%)
	Anticonvulsants	17% (14%–25%)
MSSA‐LTC score	Median (IQR) MSSA‐LTC score per facility	402 (363–435)

Abbreviations: IQR, interquartile range; MSSA‐LTC, medication safety‐self assessment for long‐term care.

### 3.2 Medication use

Residents across all facilities used a median of 13 medicines (Table 1). Over half of the residents in each facility were on polypharmacy. A higher proportion of residents were prescribed antidepressants, opioid analgesics and benzodiazepines, while anticonvulsants and antibiotics were prescribed for a lower proportion of residents.

### 3.3 Medication safety‐related processes

A high proportion of medication safety‐related processes were implemented in Australian RACFs, with a median (IQR) MSSA‐LTC score of 402 (363–435) out of a total score of 516 (Table 1). There was no association between the level of implementation of medication safety‐related processes and the RACF characteristics included in this study.

Medication safety‐related processes such as complete prescription writing by the prescriber, well‐labelling of medications and biologicals for individual residents, and single‐person use of topical preparations were the most commonly implemented processes, while routine dose adjustment of medication in residents with renal or severe hepatic impairment, use of barcoding to verify residents during medication administration, the prescriber order entry system with medication dose range checker and routine inspection and testing of electronic infusion control devices were least commonly implemented processes.

### 3.4 Association between medication safety‐related processes and medication use

**FIGURE 1:**
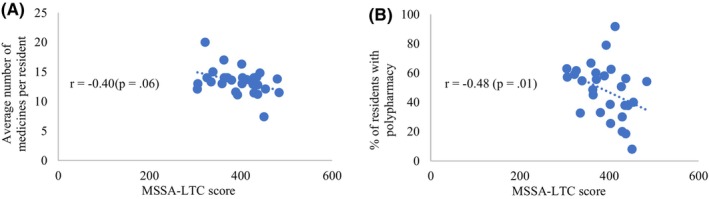
Association between the implementation of medication‐safety related processes (MSSA‐LTC score) and medication use: (A) average number of medications per resident, (B) proportion of residents with polypharmacy.

**FIGURE 2:**
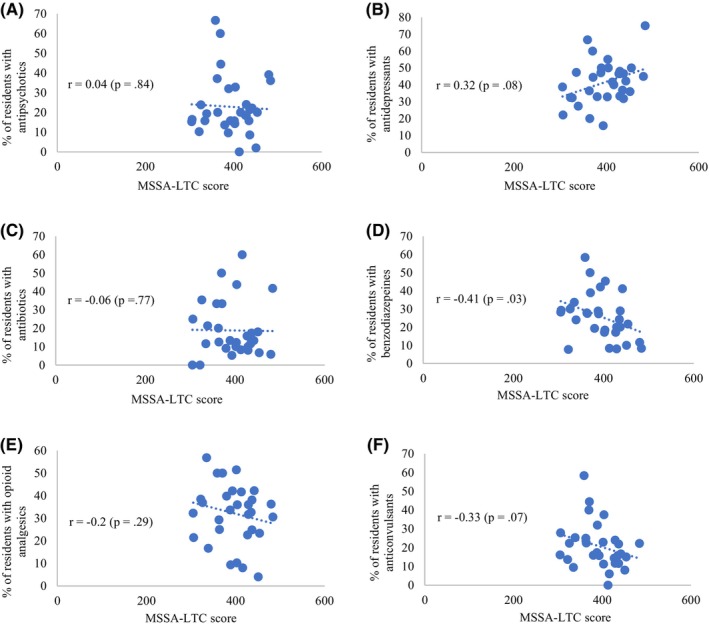
Association between the implementation of medication safety‐related processes (MSSA‐LTC score) and proportion of residents using selected medication: (A) antipsychotics, (B) antidepressants, (C) antibiotics, (D) benzodiazepines, (E) opioid analgesics, (F) anticonvulsants.

RACFs with higher MSSA‐LTC scores likely had fewer medications per resident, but this association was not statistically significant (Figure 1A). A higher facility MSSA‐LTC score was associated with a lower proportion of residents with polypharmacy (*r* = −.48, *p* = .01; Figure 1B). While looking at the individual class of medication, the higher facility MSSA‐LTC score was associated with a lower proportion of residents with one or more benzodiazepines (*r* = −.41, *p* = .03; Figure 2D). Moreover, a higher facility MSSA‐LTC score likely had lower proportion of residents using one or more opioid analgesics (Figure 2E) and anticonvulsants (Figure 2F). However, these associations were not statistically significant. In contrast, a higher facility MSSA‐LTC score was linearly associated with a higher proportion of residents using one or more antidepressants (Figure 2B), but this result was not statistically significant. There was no association between the MSSA‐LTC score and the proportion of residents with one or more antipsychotics (Figure 2A) and antibiotics (Figure 2C).

## 4 DISCUSSION

This study is the first to examine the association between the implementation of medication safety‐related processes measured by the MSSA‐LTC tool and medication use in RACFs. In general, we found that a high proportion of medication safety‐related processes were implemented in recruited Australian RACFs. We found an association between the level of implementation of medication safety‐related processes and the proportions of residents with polypharmacy and benzodiazepines, with a high facility MSSA‐LTC score associated with a lower proportion of residents with polypharmacy and benzodiazepines.

Medication safety assessment with the MSSA‐LTC tool reflected that Australian RACFs had well‐implemented and established (more mature) medication safety‐related processes. On average, facilities scored 78% of the maximum MSSA‐LTC score. Similar findings have been reported in Canada, where in a sample of 420 Canadian RACFs, the average MSSA‐LTC score was 81%.^36^ Quality use of medicine and medication safety in RACFs are high‐priority areas on Australia's national agendas, and an array of efforts have been undertaken to improve the quality use of medicines and medication safety over the last 30 years, with a broad range of policies and resources targeting health professionals, RACFs and residents.^30^ Resources, such as medication management guidelines and professional standards, were developed and updated to provide further support at the individual practitioner and facility level for improving the quality use of medicines and medication safety.^11^, ^37^ Following the Royal Commission into Aged Care Quality and Safety in 2018,^38^ the quality use of medicines and medication safety was made the 10th national health priority area by the Council of Australian Governments Health Council.^39^ All these initiatives are likely to contribute to the high level of implementation of medication safety‐related processes included in the MSSA‐LTC tool in Australian RACFs.

While overall implementation of medication safety‐related processes was high in Australian RACFs, there was variation in the level of implementation between facilities. Medication safety assessment in hospital settings using Medication Safety Self‐Assessment for Hospitals also demonstrated considerable variation in implementing medication safety‐related processes.^40^ Different RACFs have different medication safety‐related policies and procedures,^41^, ^42^ and this difference may contribute to variations in MSSA‐LTC scores. Implementing better medication safety‐related processes is the first step towards improving medication safety, and facilities with lower MSSA‐LTC scores have enormous opportunities to improve their medication safety‐related processes. The Australian Commission on Safety and Quality in Healthcare has identified four strategies for reducing healthcare variation: policies supporting optimal care, engagement of clinicians, shared decision‐making between healthcare providers and patients, and increased research into factors and outcomes associated with variation.^43^ Feedback and sharing of performance data have been used to effectively improve care within the acute care setting.^44^ Currently, Australian RACFs have access to very limited comparative data against which to examine their level of implementation of medication safety‐related possess. Our study demonstrates the potential value of assessing medication safety‐related processes to allow facilities to target their medication safety improvement activities and monitor progress over time.

This study showed an association between the level of implementation of medication safety‐related processes and medication use among residents in Australian RACFs. We found that a higher facility MSSA‐LTC score was significantly associated with lower proportions of residents with polypharmacy and benzodiazepines. Additionally, a higher facility MSSA‐LTC score was linearly correlated with a lower number of medicines per resident and a lower proportion of residents with one or more opioid analgesics and anticonvulsants. However, these results were not statistically significant. These results indicate that the level of implementation of medication safety‐related processes may influence medication use or vice versa in RACFs. Previous studies have shown that implementing different organisational initiatives to improve patient safety was associated with improved healthcare quality, safe resident care, and resident outcomes in RACFs.^45^, ^46^, ^47^, ^48^ Similarly, initiatives such as the release of prescribing guidelines, safety warnings and tightened prescribing restrictions for psychotropic prescribing seem effective in decreasing the regular antipsychotic medication use in residential aged care facilities.^21^ A recent study by Khalil et al. in an Australian hospital also suggests that a higher level of implementation of medication safety‐related processes was associated with a lower occurrence of medication errors and medication error harm.^49^ Hence, understanding the association between the maturity of medication safety‐related processes, institutional prescribing cultures and practices, and medication use may help to explore how changing underlying medication safety‐related processes could improve medication use in RACFs.

### 4.1 Strengths and limitations

This study is the first to explore the association between the implementation of medication safety‐related processes measured by the MSSA‐LTC tool and medication use in RACFs. We included RACFs from diverse geographical locations with considerable variation in facility size, rurality and ownership type. However, this study only powered to detect a moderate association between the MSSA‐LTC score and medication use at 80% power. While the convenience sample provided diversity in terms of the facilities from which data were collected, caution is required when using the results from this study to make broader inferences. Externally working pharmacists collected data on the level of implementation of medication safety‐related processes, and the chance of recall biases cannot be neglected. While associations may be described, identifying causality in this descriptive cross‐sectional study was impossible. Moreover, given the number of associations tested with such a small sample size, it might be possible that some results were due to coincidence. However, information regarding associations may help direct researchers in the subsequent phases of research on the implementation of medication safety‐related processes and medication use in RACFs.

## 5 CONCLUSIONS

This study showed that higher levels of implementation of medication safety‐related processes in RACFs, as measured with the MSSA‐LTC tool, were associated with a lower proportion of residents with polypharmacy and benzodiazepines. This study indicates that implementing medication safety‐related processes may influence medication use in RACFs. Further studies are necessary to confirm whether the level of implementation of medication safety‐related processes is associated with medication use in RACFs and to identify interventions that improve medication use in RACFs by implementing medication safety‐related processes.

## FUNDING INFORMATION

This research received no specific grant from any funding agency in the public, commercial or not‐for‐profit sectors.

## CONFLICT OF INTEREST STATEMENT

No conflicts of interest declared.

## Data Availability

The data sets generated during and/or analysed during this study are available from the corresponding author upon reasonable request.
